# Amine oxidase 3 is a novel pro-inflammatory marker of oxidative stress in peritoneal endometriosis lesions

**DOI:** 10.1038/s41598-020-58362-3

**Published:** 2020-01-30

**Authors:** Marie-Laëtitia Thézénas, Bianca De Leo, Alexis Laux-Biehlmann, Cemsel Bafligil, Bernd Elger, Thomas Tapmeier, Karl Morten, Nilufer Rahmioglu, Stephanie G. Dakin, Philip Charles, Fernando Estrada Martinez, Graham Steers, Oliver M. Fischer, Joerg Mueller, Holger Hess-Stumpp, Andreas Steinmeyer, Sanjiv Manek, Krina T. Zondervan, Stephen Kennedy, Christian M. Becker, Catherine Shang, Thomas M. Zollner, Benedikt M. Kessler, Udo Oppermann

**Affiliations:** 1 Target Discovery Institute, Nuffield Department of Medicine, University of Oxford, Oxford, OX3 7FZ UK; 2 Bayer AG, R&D, Gynaecological Therapies, 13342 Berlin, Germany; 3 Botnar Research Centre, NIHR Biomedical Research Unit Oxford, Nuffield Department of Musculoskeletal Sciences, University of Oxford, Oxford, OX3 7LD UK; 4 Endometriosis CaRe Centre, Nuffield Department of Obstetrics & Gynaecology, Oxford, UK; 5 Wellcome Trust Centre for Human Genetics, University of Oxford, Oxford, OX3 7BN UK; 6 Freiburg Institute for Advanced Studies (FRIAS), University of Freiburg, 79104 Freiburg, Germany

**Keywords:** Infertility, Chronic inflammation

## Abstract

Endometriosis is a common gynaecological disease of women in reproductive age, and is thought to arise from retrograde menstruation and implantation of endometrial tissue, mostly into the peritoneal cavity. The condition is characterized by a chronic, unresolved inflammatory process thereby contributing to pain as cardinal symptom in endometriosis. Elevated reactive oxygen species (ROS) and oxidative stress have been postulated as factors in endometriosis pathogenesis. We here set out for a systematic study to identify novel mechanisms and pathways relating to oxidative stress in ectopic peritoneal lesions. Using combined proteomic and transcriptomic approaches, we identified novel targets including upregulated pro-oxidative enzymes, such as amine oxidase 3/vascular adhesion protein 1 (AOC3/VAP1) as well as downregulated protective factors, in particular alkenal reductase PTGR1 and methionine sulfoxide reductase. Consistent with an altered ROS landscape, we observed hemoglobin / iron overload, ROS production and lipid peroxidation in ectopic lesions. ROS-derived 4-hydroxy-2-nonenal induced interleukin IL-8 release from monocytes. Notably, AOC3 inhibitors provoked analgesic effects in inflammatory pain models *in vivo*, suggesting potential translational applicability.

## Introduction

Endometriosis, affecting 5–10% of women in reproductive age, is a gynaecological disease characterized by the presence of ectopic endometrial tissue most often found in the pelvic cavity^1,2^. Retrograde menstruation is a widely accepted pathogenic mechanism for the formation of peritoneal lesions, in which uterine epithelial and stromal cells are disseminated during menstruation and implanted into the peritoneal cavity via the Fallopian tubes^3,4^. Peritoneal lesions are macroscopically classified based on colour, appearing as white, red, brown, or black lesions. This appearance seems to be determined by endometrial gland content and a variable adjacent stromal reaction, likely reflecting a chronicity process rather than different levels of disease activity^5^. Additional contributors to disease such as genetic, epigenetic and environmental factors exist, since a majority (90%) of women undergo retrograde menstruation associated with successful clearance of menstrual debris from the peritoneal cavity^3,6–9^. However, the formation of ectopic endometrial lesions is a signature event that leads to the hallmarks of disease, comprising sustained inflammation, severe and chronic pain, as well as subfertility^10,11^.

The dependency of eutopic endometrium and ectopic lesions on oestrogen levels results in the cyclic growth, shedding and bleeding of ectopic lesions, which in turn elicits an inflammatory response, characterized by infiltration of immune cells and accompanied by a pro-inflammatory cytokine profile^8^. As a consequence of lesion bleeding, iron overload has been described in different components of the peritoneal cavity, such as peritoneal fluid (PF), endometriotic lesions, peritoneum, as well as in infiltrating monocytes and macrophages^12^. Consequently, increased amounts of iron and increased heme catabolism may promote oxidative stress, which is considered to play a key role in the pathophysiology of endometriosis^13^. Indeed, oxidative stress resulting in lipid peroxidation products (LPPs) such as 4-hydroxy-2-nonenal (4-HNE) and malondialdehyde (MDA) has been documented in the stroma of ovarian endometriotic tissue and in the PF of endometriosis patients^14–17^.

Moreover, studies have proposed a link between oxidative stress and pain through inflammation in endometriosis, pointing towards dysregulated immunomodulation. Increased cytokine concentrations produced by stimulated peritoneal macrophages have been reported in endometriosis patients^8,18^. This inflammatory state can stimulate growth and activation of nerve fibres, leading to additional activation of pain pathways^19^.

Macrophage infiltration of endometriotic lesions may be involved in pelvic pain and inflammation associated with endometriosis^8,18^. In the context of the cascade of events causing pain, failure to detoxify LPPs could enhance pain sensation in endometriosis patients. For example, LPP detoxifying enzymes such as aldehyde dehydrogenase 2 (ALDH2) could possibly shield the organism from endogenously damaging LPPs arising from lipid peroxidation under oxidative stress^20–22^. This is indirectly supported by epidemiological evidence describing an ALDH2 polymorphism in Asians, pointing towards a potential role of ALDH2 in pain prevalence and pain tolerance, threshold, or intensity^23–25^. Furthermore, a sustained cycle of inflammation and oxidative stress facilitates the implantation of ectopic endometrium^26^, supporting a central role of oxidative stress in the pathogenesis of endometriosis. Akin to malignant tumour growth, neovascularization of ectopic lesions is a prerequisite for successful implantation of endometrial cells and is accomplished through factors such as vascular endothelial growth factor (VEGF), secreted by stromal and infiltrating immune cells^27^.

Proteomic approaches have been used to identify possible biomarkers correlating with endometriosis pathology, mostly in serum or PF^2,28,29^. Besides critical cytokines and growth factors such as interleukin IL-1β, IL-6, IL-10 or VEGF, these studies have also identified alterations in indicators of oxidative stress, such as paraoxonase, superoxide dismutase (SOD), thioredoxin binding protein, and others^30^, again lending support to the hypothesis of oxidative stress as a hallmark of disease. Our interest in target discovery in endometriosis now instigated a systematic proteomic approach, further supported by transcriptomics, to identify in greater depth the potential underlying reasons for upregulated oxidative stress and its possible consequences.

## Results

### Oxidative protein modifications are increased in ectopic lesions

**Figure 1:**
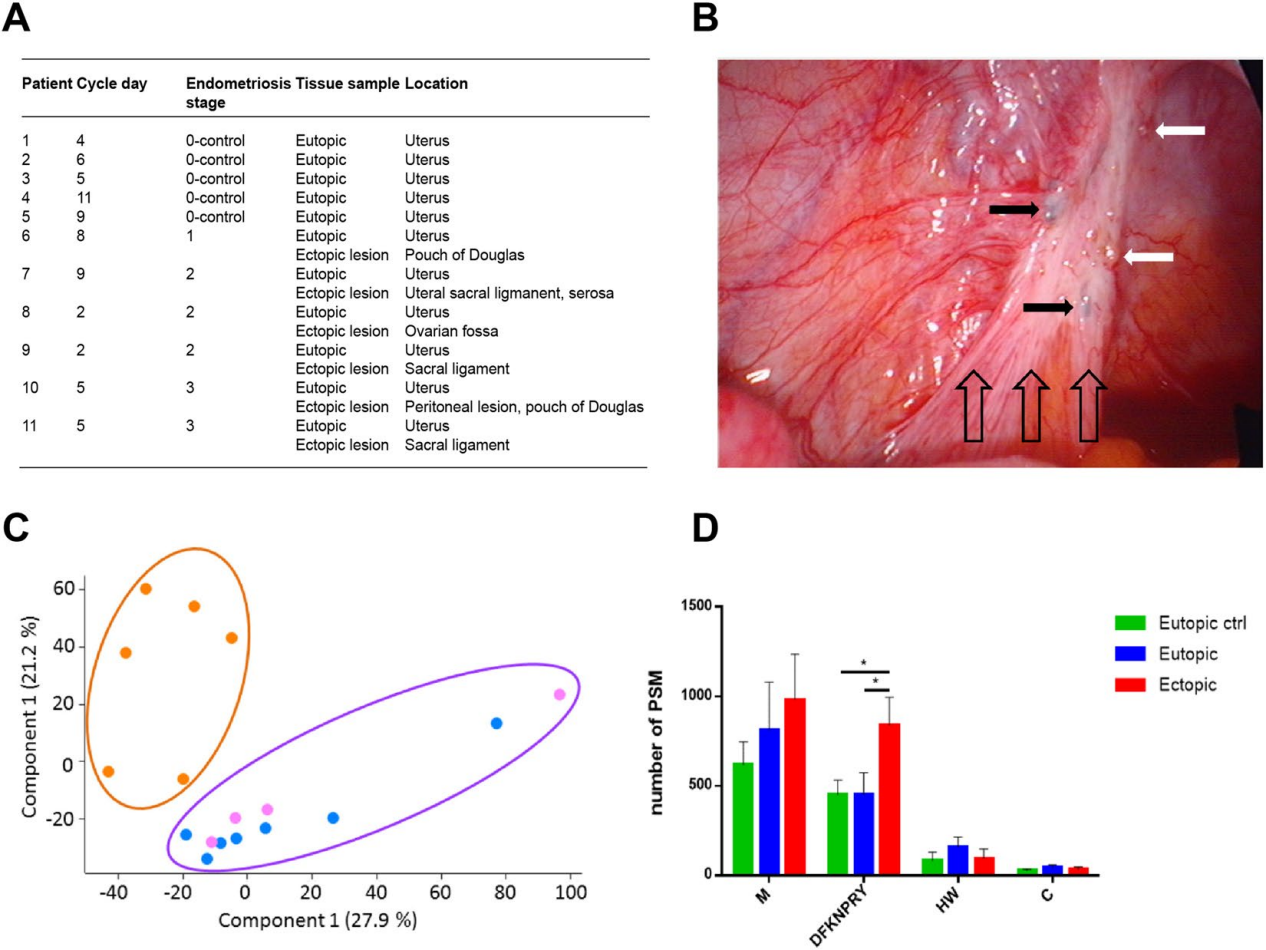
Proteomic analysis identifies oxidative protein modification in ectopic lesions. (**A**) Patient demographics and samples used in the proteomic analysis. (**B**) Example of peritoneal endometriotic lesions. The black filled arrows indicate blue lesions, the white filled arrows red lesions. Note the inflammation and fibrosis (transparent arrows). (**C**) Principal component analysis (PCA) separates endometriotic lesion from control tissue samples. The panel shows clustering of eutopic endometrium from control (blue) and endometriosis patients (magenta) against peritoneal lesions (yellow). (**D**) Oxidative protein modifications identified by MS in eutopic and ectopic samples. Modifications are grouped according to amino acid residue or N/K-linked formylation (M-methionine, D-aspartate, F-phenylalanine, K-lysine, N-asparagine, P-proline, R-arginine, Y-tyrosine, H-histidine, W-tryptophan and C-cysteine).

Paired tissue biopsies (eutopic endometrium, ectopic lesion) of patients (Fig. 1A,B) with confirmed endometriosis were subjected to a shotgun proteomic approach leading to identification of 2307 proteins. To further validate results, eutopic endometrium from control patients (confirmed non-endometriosis diagnosis) was included in the study. Principal component analysis (PCA) revealed that ectopic samples clustered differently from eutopic samples, irrespective of control and disease cohorts (Fig. 1C). No difference was observed between normal endometrium from controls and from eutopic endometrium in endometriosis patients, suggesting that the implantation of endometrium and development of lesions leads to a significant change in the proteome.

We first investigated possible differences in oxidative protein modifications in lesions and eutopic endometrium as a consequence of ROS. Analysis of peptide spectral matching data revealed higher levels of oxidative modifications in proteins derived from ectopic lesions (Fig. 1D). Significantly higher (p < 0.001) levels of oxidative modification of aspartic acid, phenylalanine, lysine, asparagine, proline, arginine and tyrosine residues were observed in eutopic samples from endometriosis patients. Although not statistically significant (p > 0.05), oxidized methionine residues also showed a tendency to higher levels in endometriosis samples (ectopic > eutopic) when compared to control. Levels of histidine, tryptophan and cysteine modifications showed no significant difference between ectopic lesions and controls, and oxidation and formylation levels for histidine, tryptophan and cysteine were generally low in ectopic lesions and controls.

### The lipid peroxidation product 4-HNE is present in ectopic lesions and induces IL-8 secretion from monocytes

**Figure 2:**
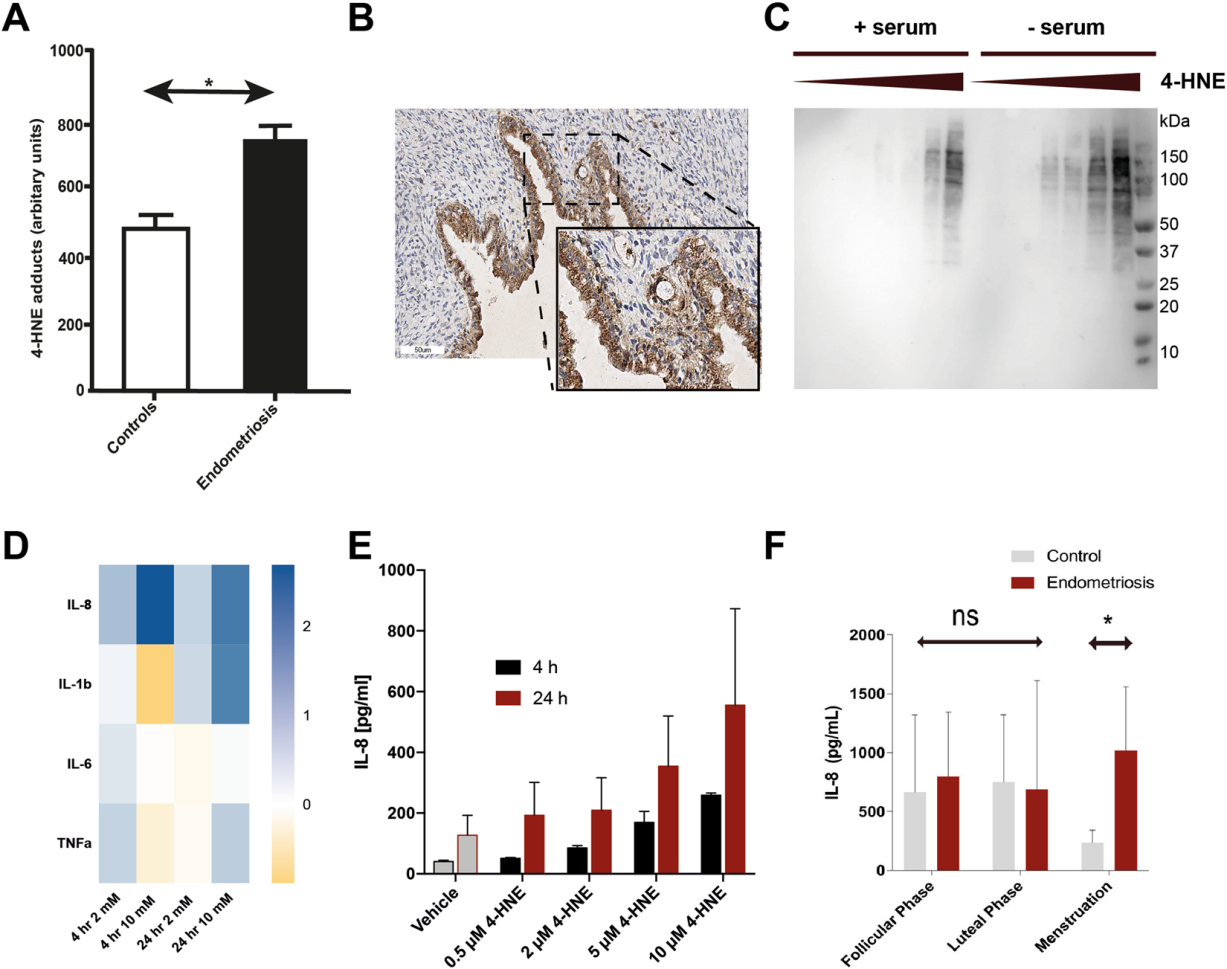
4-hydroxynonenal (4-HNE) modifications are increased in endometriotic lesions and lead to an increase of IL-8 secretion in human monocytic cells. (**A**) 4-HNE modifications in peritoneal fluid are increased in endometriosis *vs*. control patients. (**B**) IHC of 4-HNE modifications in ectopic lesions. (**C**) Western blot showing dose-dependent increase of 4-HNE adducts of treated THP-1 monocytic cells. (**D**) Heat map showing cytokine profiles from PBMC at two 4-HNE concentrations (2, 10 μM) and 4 hr and 24 hr time points indicating IL-8 response. Scale indicates log-2 fold changes. (**E**) Dose-response of IL-8 secretion in PBMC. (**F**) IL-8 levels in peritoneal fluid between endometriosis and control patients, separated by menstrual cycle phase. Abbreviations ns = not significant, *p < 0.05.

Using Western blotting, higher levels of ROS-mediated protein adducts (4-HNE adducts) were observed in peritoneal fluid (Fig. 2A) of endometriosis patients. These 4-HNE modifications were also widespread in ectopic lesions (Fig. 2B). To investigate functional consequences of ROS modification on immune cells, we used the human monocytic cell line THP-1. Dose-dependent increases of 4-HNE adducts were apparent when treated with 4-HNE in the presence or absence of serum (Fig. 2C). This 4-HNE treatment also leads to changes in cytokines, measured in the culture supernatant upon stimulation (Fig. 2D). With the exception of IL-1β at higher 4-HNE concentrations (10 μM) at extended incubation (24 hours), levels of secreted IL-1β, IL-6 or TNFα only changed slightly compared with basal levels of vehicle-treated cells, at intermediate (2 μM) or high concentrations (10 μM) of 4-HNE at 4 and 24 hours. In contrast, IL-8 secretion displayed a robust and significant dose-dependent response, also observed in human peripheral blood mononuclear cells (PBMCs) (Fig. 2E) suggesting that increased IL-8 secretion is a physiological response of 4-HNE exposure to monocytes. Exposure of THP-1 cells to 4-HNE not only increased protein adduct formation, but also cell death in a dose-dependent fashion (SI Fig. 1), where 10 μM of 4-HNE (as used in experiments detailed above) appeared to be the maximal dose that did not cause excessive cell death in serum-positive and –negative conditions. Importantly, we observed that during the menstrual phase, IL-8 levels in control patients dropped compared to luteal and follicular stages, whereas in endometriosis patients, chemokine levels remained significantly elevated (Fig. 2F). Although ROS levels were not measured in this particular arm of our study, this observation suggests a potential pathophysiological mechanism in endometriosis, and in general confirms the previous observation of elevated IL-8 levels in endometriosis^31^.

### Oxidative stress and imbalances in protective metabolic pathways are hallmarks of endometriotic lesions

**Figure 3:**
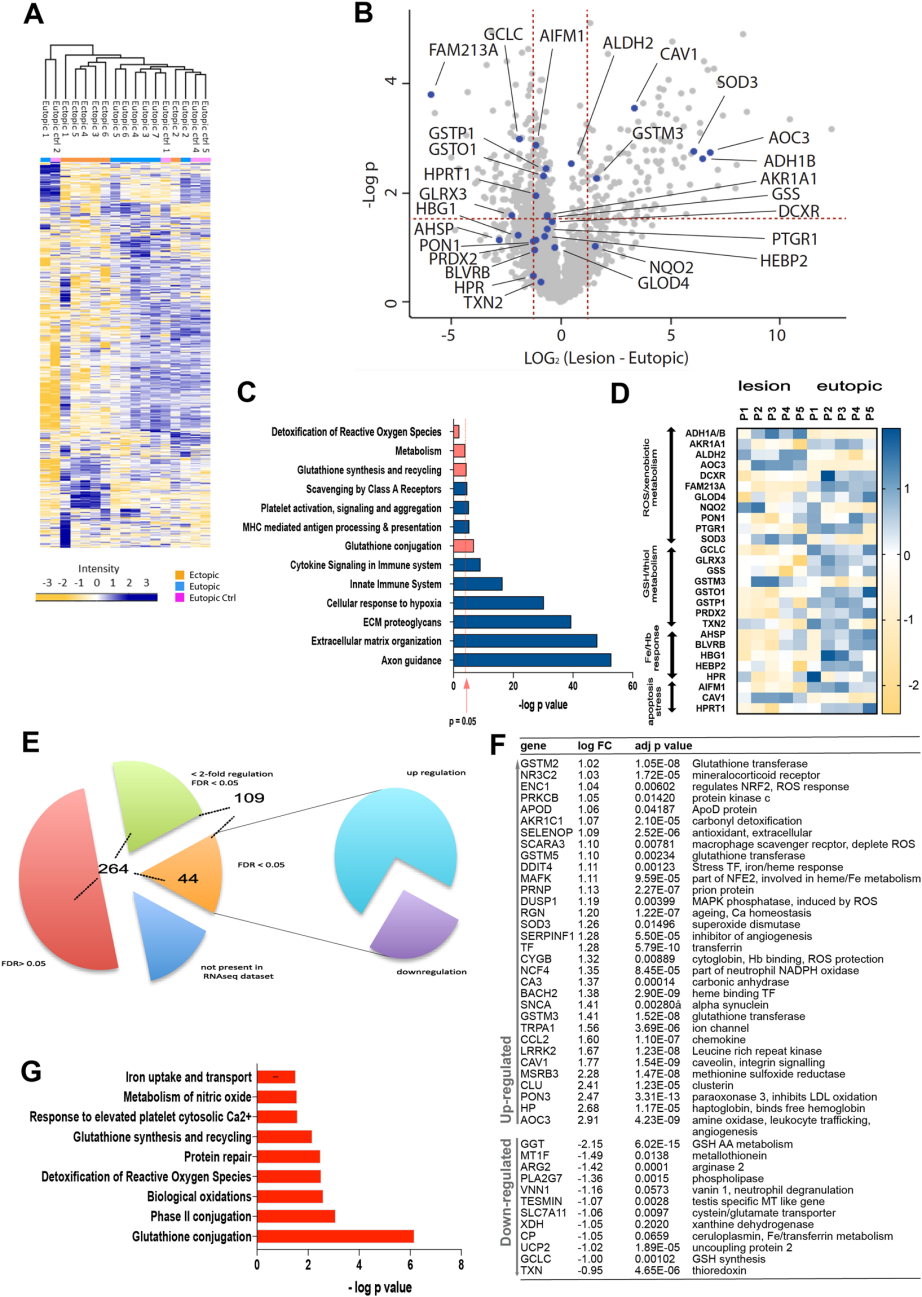
Proteomic and transcriptomic data reveal oxidative stress, lipid peroxidation and iron uptake signatures in endometriotic lesions. (**A**) Heat map of statistically significant protein abundance profiles identified in the proteomic analysis of ectopic peritoneal lesions, eutopic control and endometriosis. (**B**) Volcano plot showing the differential abundance of each protein identified by MS/MS according to the Log2 (difference lesion-eutopic) (x axis) and the –log_10_ p-value (y axis) using a Student t-test. Data was generated using 2307 quantified proteins to compare eutopic *vs*. ectopic (lesions) tissues (n = 6 per group). Squares above the red dotted lines represent the proteins significantly different (p-value < 0.05) and a >2-fold difference between the ectopic and eutopic samples. Proteins highlighted in blue are the same as shown in Fig. 4D. (**C**) Reactome pathway analysis of significantly differently expressed proteins between eutopic and ectopic patient tissues. A binomial test was used to calculate the probability and the p-values were corrected for multiple testing (Benjamini-Hochberg). (**D**) Heat map of selected genes involved in ROS/xenobiotic metabolism, GSH and thiol metabolism, Fe/Hb response and apoptosis or stress. Colour scale indicates z-score values (see also SI Data 1). (**E**,**F**) Identification strategy and list of oxidative stress/lipid peroxidation genes expressed >2-fold or <0.5-fold in RNAseq dataset. Numbers in (**E**) indicate categorized genes (see main text). (**G**) Assignment of significantly different proteins identified in the RNAseq experiment to sub-pathways associated with oxidative stress and lipid peroxidation using the Reactome database.

The proteomic analysis identified 2307 proteins with a false discovery rate (FDR) of 1% (SI Data 1). We identified 581 proteins showing significant differences in abundance between ectopic lesions and eutopic endometrium using a paired Student t-test (Fig. 3A,B, SI Data 1). Biological categories in which these proteins are significantly enriched comprise axon guidance, extracellular matrix organisation, immune system and metabolic pathways such as glutathione (GSH) conjugation. The identification of the latter pathways in lesions led us to investigate further metabolic proteins in relation to ROS generation and lipid peroxidation pathways (Fig. 3C). We identified 27 proteins in the proteomic data set (Fig. 3D) that were related to these pathways. Although not all proteins reached stringent statistical significance in this dataset, likely due to the limited samples numbers, the data indicate that a number of metabolic proteins involved in ROS or lipid peroxidation and cell death is differentially regulated in lesions as compared to their matched controls. Among the metabolic proteins upregulated in lesions, alcohol dehydrogenase 1 (ADH1), quinone oxidoreductase 2 (NQO2), superoxide dismutase 3 (SOD3) and glutathione S transferase μ3 (GSTM3) are involved in detoxification of xenobiotic alcohols, ROS and its derived reactive carbonyl such as hydroxyalkenals (e.g. 4.HNE) or MDA. Conversely, several oxidative stress-related enzymes and factors were expressed at lower levels in lesions. These included enzymes such as paraoxonase (PON), alkenal reductase PTGR1, enzymes involved in thiol and glutathione metabolism such as glutamate cysteine ligase (GCLC), glutathione synthetase (GSS), glutathione S transferases (GSTO and GSTP1), peroxiredoxin (PRDX2), alpha haemoglobin stabilising protein (AHSP), biliverdin reductase (BLVRB), hypoxanthine phosphoribosyl transferase (HPRT1), apoptosis inducing factor mitochondria 1 (AIFM1), FAM213 and glyoxalase domain containing 4 (GLOD4). Of particular interest was the identification of the upregulated copper-dependent amine oxidase AOC3 (also known as vasculature adhesion protein 1, VAP-1), which catalyses the oxidative deamination of primary amines, producing the corresponding aldehyde, hydrogen peroxide and ammonium^32^, hence could significantly increase levels of ROS and lipid peroxidation products within lesions. Another novel upregulated protein identified in the proteomic dataset (Fig. 2A,B) was caveolin 1 (CAV1), an integral component of caveolae lipid rafts in plasma membranes, previously shown to mediate cellular senescence and IL-6 signalling in fibroblast^33^. Although highly informative, we hypothesised that the mass spectrometry approach delivered only a fraction of markers involved in dysregulation of oxidative stress. It was therefore of interest to compare the proteomic subset against a transcriptomic dataset, obtained from a separate collection of matched samples of eutopic endometrium and ectopic lesions. Using a shortlist of 308 genes (SI Data 2) considered being involved in lipid peroxidation and oxidative stress pathways identified through literature searches and the GLAD4U search engine^34^ we analysed an RNAseq dataset of matched endometrial eutopic and ectopic lesion samples derived from 18 endometriosis patients at different disease stages (SI Data 3). Out of 17,673 genes present in the RNAseq data, 264 of the selected 308 genes had expression data; 109 (41%) showed a genome-wide false discovery rate (FDR) corrected p-value < 0.05 for differential expression compared to n = 4,897 (27%) of all 17,673 genes (Fig. 3E). Out of these 109, 44 showed the largest differential expression (<0.5 or >2-fold) in the ectopic samples compared with the eutopic samples (Fig. 3E,F). Importantly, a look-up of genes assigned to these categories (lipid peroxidation and oxidative stress) confirmed ROS detoxification, GSH conjugation and iron/heme overload pathways as differentially regulated between ectopic lesions and eutopic endometrium (Fig. 3G), in addition to pathways such as protein repair, which had previously not been identified in the proteomic dataset. Of note, several genes such as SOD3, GSTM3, AOC3, CAV1, gamma-glutamyltransferase (GGT) and GCLC overlap between the transcriptomic and proteomic datasets, providing a significant correlation between these two approaches.

### Ectopic lesions are characterized by increased iron and heme overload, myeloid infiltration, oxidative stress, phagocytosis and protein repair

**Figure 4:**
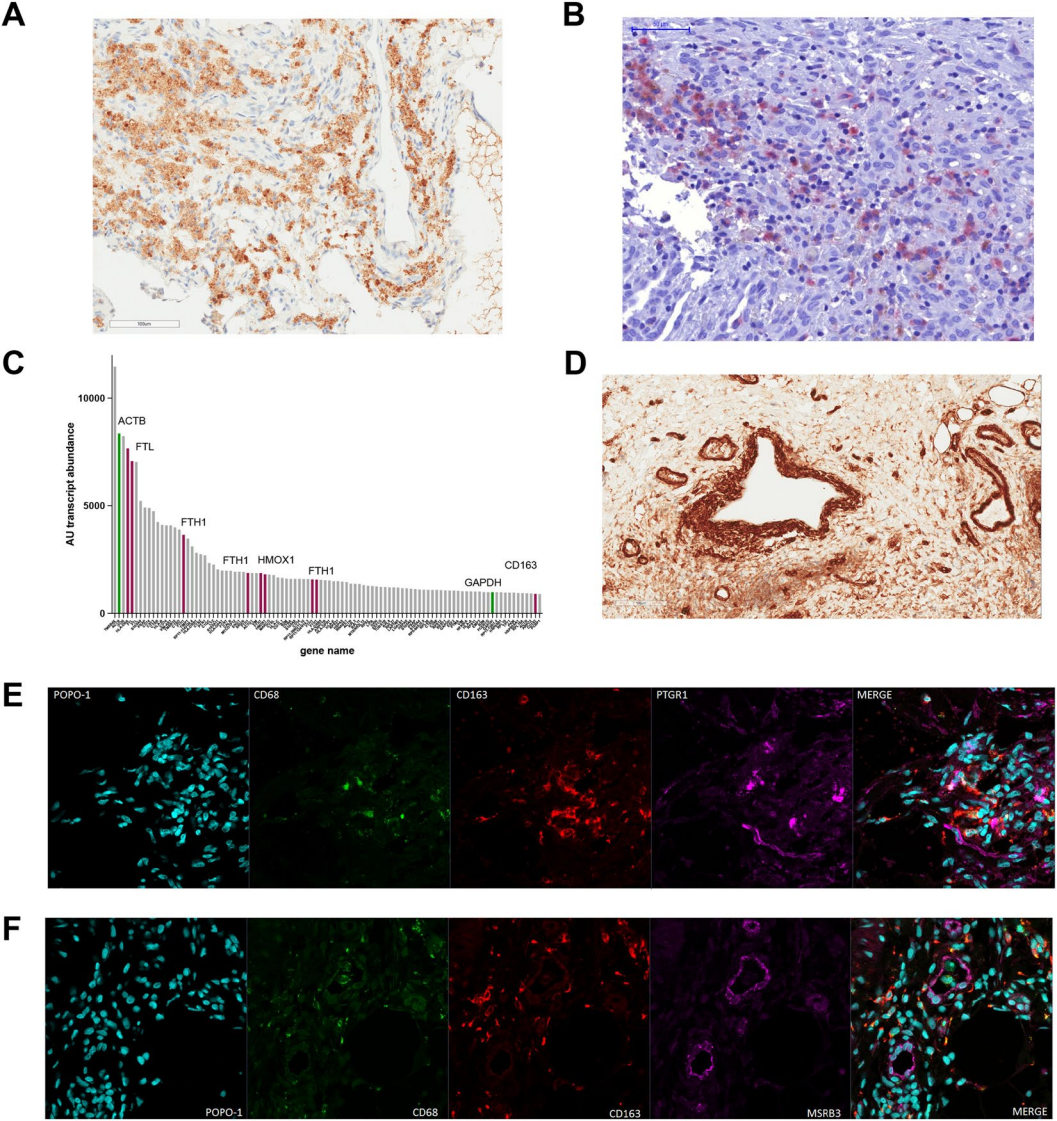
Hemoglobin, iron overload, ROS production and lipid peroxidation in ectopic lesions. (**A**,**B**) Immunohistochemistry staining of ectopic lesions showing (**A**) haptoglobin expression, (**B**) infiltrating CD68^+^ macrophages in a typical lesion. (**C**) Ranking plot of top 100 expressed transcripts derived from RNAseq of peritoneal macrophages of endometriosis patients. Red bars indicate transcripts involved in iron/heme metabolism, green bars display housekeeping genes such as beta-actin (ACTB) and GAPDH. (**D**) Immunohistochemistry of AOC3 in ectopic lesions. (**E**,**F**) Co-localisation studies of alkenal reductase PTGR1 (**E**) and methionine sulfoxide reductase MSRB3 (**F**) (both pink) in comparison to infiltrating macrophages (CD68 green, CD163 red). Popo1 staining is used to highlight nuclei.

Although no clearly dysregulated levels of heme or iron-related proteins were identified in ectopic lesions in the proteomic dataset, the instigating event in the inflammatory process appears to be the deposition of menstrual debris and erythrocyte-derived heme and iron as shown previously^12^, which is also supported by our RNAseq data. The observed increased expression of haptoglobin (HP), transferrin (TF), cytoglobin (CYGB) and the heme-responsive master transcription factor BACH2, clearly indicate an iron-heme overload response in lesions (Fig. 3F,G). Immunohistochemical staining for iron and heme scavenging proteins such as haptoglobin (Fig. 4A), transferrin or BACH2 (SI Figs. 2 and 3), show that expression in lesions is not restricted to glandular epithelial structures as seen in eutopic endometrium, but occurs throughout the stroma in the vicinity of lesions, indicating a more generalized response.

Ectopic lesions are characterized by an infiltration of CD68+ and CD163+ macrophages (Fig. 4B). The expression profile of these phagocytes shows a characteristic and strong transcriptional heme/iron response, supported by RNAseq data of peritoneal macrophages derived from endometriosis patients undergoing laparoscopy for lesion excision (Fig. 4C). Among the top 100 expressed transcripts in these macrophages, various transcripts for ferritin light and heavy chain (FTH1, FTL), heme oxygenase 1 (HMOX), and CD163, the heme scavenger receptor, were identified. Taken together, the IHC and RNAseq analyses clearly highlight that iron and heme overload signatures, as well as phagocytic responses, are key elements in ectopic lesions. Whilst infiltrating myeloid cells constitute a fraction of cells found in ectopic lesions and contribute to oxidative stress levels, it appears that ROS generation and inactivation, tissue modification and repair mechanisms are not necessarily restricted to a given cell type or structure within the lesions. We used immunohistochemistry and immunofluorescence microscopy to study the expression of proteins involved in ROS generation, defence and repair (Fig. 4D–F, SI Figs. 2 and 3). The pro-oxidative amine oxidase AOC3, identified in the proteomic and transcriptomic work is strongly expressed throughout ectopic lesions with highest levels found around glandular epithelia or vasculature (Fig. 4D). Enzymes like the alkenal reductase PTGR1 are involved in inactivation of ROS-derived LPPs such as 4-HNE or MDA^35^ and its expression is noted across stroma and glands (Fig. 4E). Another protective, oxidative damage repair enzyme like methionine sulfoxide reductase MSRB3^36^ (Fig. 4F) is found solely in the epithelial structures of ectopic glands. Taken together, these experiments highlight distinct differences in intra-lesion distribution of proteins involved in generation and inactivation of ROS products and adducts.

### Inhibition of pro-oxidative AOC3 leads to analgesic effects *in vivo*

**Figure 5:**
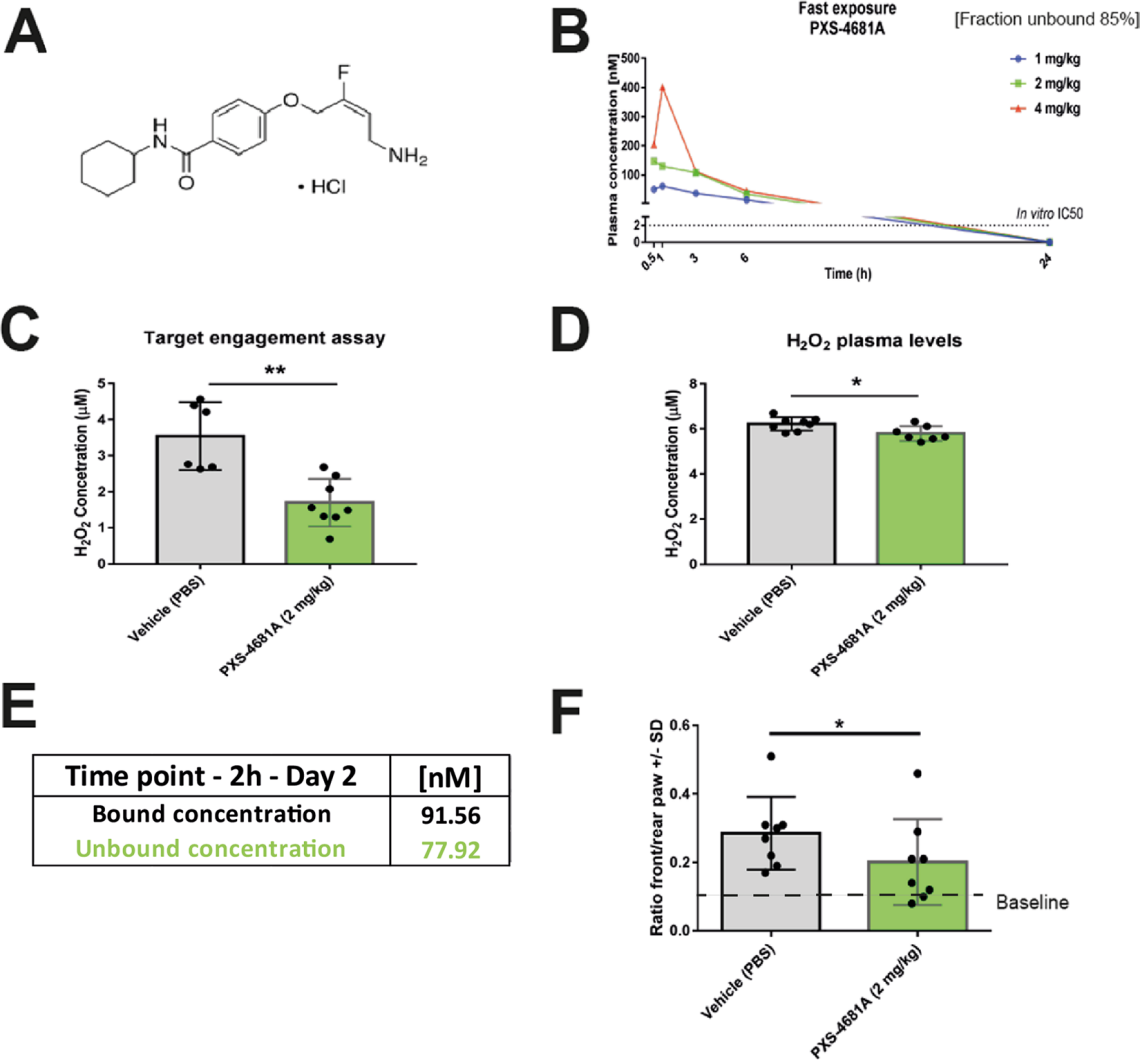
AOC3 inhibitor PXS-4681A shows analgesic effects in the endometriosis inoculation mouse model. (**A**) Structure of AOC3 inhibitor PXS-4681A, orally administered BID at 2 mg/kg. (**B**) Unbound plasma levels of PXS-4681A (at 1-2-4 mg/kg). (**C**) Target engagement results (2 mg/kg). (**D**) Changes in H_2_O_2_ in plasma. (**E**) Plasma exposure of PXS-4681A at day 2. (**F**) Front/rear paw ratio measure using the dynamic weight bearing system indicating reduction of pain behaviour under treatment.

The identification of AOC3 in the proteomic and transcriptomic experiments, along with the strong immunoreactivity found in lesions, prompted us to further investigate the role of the enzyme in an endometriosis inoculation model. We hypothesized that inhibition of AOC3 would blunt the consequences of a pro-inflammatory and pro-oxidative stimulus such lesion inoculation, and we were interested if this would lead to an analgesic effect. We used the previously described compound PXS-4681A^37^ (Fig. 5A) in an inoculation endometriosis model. Animals were subjected to 2 mg/kg BID dose of the inhibitor, which resulted to be above *in vitro* IC_50_^37^ in the fast exposure test (Fig. 5B), starting 1 hr before intraperitoneal injection of uterine punches (12×/animal). At day 2, hydrogen peroxide production was measured in plasma, and as target engagement test in white gonadal fat, showing a significant reduction in systemic H_2_O_2_ levels compared to vehicle treated controls (Fig. 5C,D). Furthermore, animals showed reduced pain-related spontaneous behaviours as measured in front/rear paw ratio in the dynamic weight bearing assay^38^ and rearing time (Fig. 5F), suggesting that AOC3 inhibition is leading to a reduction of oxidative stress and pain in the inoculation model.

## Discussion

Studies addressing the pathological characteristics of endometriosis have revealed a vicious cycle of oxidative stress generated by high levels of ROS that in turn facilitates the implantation of the ectopic endometrium^26^. Our data support a previously postulated pathomechanistic scenario^12–17,39^, whereby retrograde menstruation leads to implantation of endometrium in the peritoneal cavity, which in turn generates a sustained pro-inflammatory environment^11^. This is characterised by increased iron and heme overload through ectopic menstruation and attempted clearance through infiltrating myeloid phagocytes. Although the patient collectives used for this study are small and could not be further stratified in respect to metadata like BMI or cycle phases, both of which can impact phenotypic markers, our results highlight several novel aspects in the context of the pathophysiology of endometriosis.

First, the results not only confirm known differences in ROS and heme pathways between ectopic and eutopic endometrium, but also significantly extend and identify novel elements, including pro-apoptotic and protein repair mechanisms. We show that several metabolic and oxidative stress pathways and ROS/lipid peroxidation products are differentially regulated and affected in endometriosis *versus* normal eutopic tissue. The identification of the copper-dependent amine oxidase 3 as a major distinguishing factor between eutopic and ectopic endometrium indicates two possible roles in endometriosis pathology. As a by-product of amine oxidation, AOC3 produces hydrogen peroxide, which can directly increase ROS levels. Furthermore, the adhesion properties of the enzyme^32^ facilitate endothelial leukocyte adhesion and transmigration, consequently enabling immune cell infiltration. It is therefore tempting to speculate that AOC3 overexpression in endometriosis lesions contributes to a local permissive inflammatory environment favouring lesion generation and implantation, akin to the role of this pro-inflammatory enzyme in myeloid cell recruitment in metastasis or lymphocyte recruitment in a variety of human pathologies^32,40,41^.

Failure to detoxify LPPs could enhance pain sensation in endometriosis patients. Although we detect LPP detoxifying enzyme systems such as glutathione S-transferases or ALDH2, significant levels of 4-HNE adducts were observed in ectopic lesions and eutopic endometrium, consistent with previous observations of ROS and endometriosis^20–22^. The observed increased levels of ROS and LPP-derived oxidative protein modifications are accompanied by lower levels in lesions of LPP-inactivating enzymes involved in GSH synthesis such as GCLC, or protective enzymes like glutaredoxins or peroxiredoxins. This is evidence that lesions are characterised by an unbalanced set of protective measures and could explain a vicious cycle of sustained elevated ROS levels, possibly increased apoptosis levels, and changes in inflammatory properties of lesions through altered protein modifications.

Second, we describe a relationship between oxidative stress, IL-8 and angiogenesis in endometriosis that furthers our understanding of the pathophysiology of the disease. Similar to tumour metastases, endometriotic implants require neovascularization to proliferate and invade ectopic sites within the host. Endometrial tissue is a rich source of pro-angiogenic factors, including VEGF, which has emerged as critical vasculogenic regulator in endometriosis, and which may be expressed by infiltrating immune cells^27^. Elevated ROS levels have been shown to trigger Toll-like receptor, NF-κB, and HIF1α pathways, which can result in increased angiogenesis^42,43^. Various inflammatory agents, growth factors, and oxidative stress activate NF-κB, which has been linked to cellular transformation, proliferation, apoptosis, angiogenesis, and invasion^44^. The data obtained indicate that oxidative stress and LPPs alter the cytokine profile of myeloid cells, in particular IL-8 secretion, as driver of myeloid infiltration and lesion vascularization. We demonstrate that at physiological doses of 4-HNE exposure, human monocytic THP-1 cells increase IL-8 secretion, protein adduct formation, and cell death. Interleukin-8 is important in regulating inflammatory cytokine cascades in endometriosis, but also appears to constitute a forward loop in the ROS-inflammation axis. Accordingly, monocytes and macrophages as generators of inflammatory cytokines are potentially involved in the 4-HNE oxidative cascade in endometriosis and can also contribute to myeloid infiltration and vascularization of ectopic lesions through IL-8 production. The angiogenic properties of this chemokine have long been recognized, particularly in oncology^45^, and also in endometriosis^46^. The clinical data from us and others that IL-8 is significantly elevated in the PF of women with endometriosis^31^ and associated with pain^47^, further supports the observation that this cytokine promotes the disease in a dose-dependent manner^48,49^.

It has been suggested that therapeutic approaches that target the restoration of normal levels of ROS in the peritoneum may therefore be possible candidates in the treatment of endometriosis or reduction of its symptoms^26^. And indeed, by identifying the novel pro-oxidative lesion marker AOC3 in peritoneal lesions, and by knowing its beneficial effect on inflammatory pain using a small molecule inhibitor approach^50^, we here provide pre-clinical proof of concept that manipulation of the pro-oxidative AOC3 mechanism in the peritoneal environment leads to a significant reduction of inflammatory pain, the cardinal symptom in endometriosis.

## Materials and Methods

### Patient samples

Patient studies were carried out in accordance to guidelines under the ENDOX study and all experimental protocols were approved by the local Research Ethics Committee (National Health Services (NHS) Research (NRES) Committee South Central-Oxford) (09/H0604/58). Informed written consent was provided by patients participating in the study, and samples were collected from patients undergoing laparoscopy at the John Radcliffe Hospital, Oxford. In all cases diagnosis was confirmed surgically and by histology. For the proteomic and transcriptomic arms of the study distinct patient populations were used and further collection details are provided in the SI material.

### Protein extraction and sample preparation

Tissue material was homogenised and extracted, protein extracts were subjected to in-solution trypsin digestion and data processed as described^51^.

### Transcriptome analysis

Illumina compatible next-generation sequencing libraries were prepared and sequenced from ribosomal RNA depleted or poly A+ RNA samples using standard protocols and sequenced on HiSeq. 2500 or NextSeq. 500 platforms.

### Cell culture

Human peripheral blood or peritoneal fluid cells were isolated by Ficoll centrifugation, cultured in the presence of foetal calf serum (20%) or as indicated, and treated as described in the results sections.

### *In vivo* endometriosis inoculation model

The experimental procedures, housing, treatments, and analysis of the animals were performed in accordance with policies and directives of LAGeSo (Landesamt für Gesundheit und Soziales Berlin; Germany) and the study was approved by the internal review board at Bayer AG.

### Statistical analysis

If not stated otherwise, data are represented as the mean ± SD of least three independent experiments. Statistically significant differences of *in vitro* data were identified with Student t-tests, one-way or two-way ANOVA (with Bonferroni´s multiple comparison test), as indicated in the figure legends. Patient data were analysed as indicated in the figure legends and corresponding experimental sections.

A detailed description of experimental procedures regarding cell culture, immunofluorescence and immunohistochemistry staining, quantitative mass spectrometry-based proteomics, *in vivo* experiments, and analysis of patient data can be found in Supplementary Information.

## Supplementary information

SI Appendix.

Dataset 1.

Dataset 2.

Dataset 3.

## Data Availability

The mass spectrometry proteomics data have been deposited to the ProteomeXchange Consortium via the PRIDE partner repository with the dataset identifier PXD006553.
