# Author Correction: Amine oxidase 3 is a novel pro-inflammatory marker of oxidative stress in peritoneal endometriosis lesions

**DOI:** 10.1038/s41598-020-62492-z

**Published:** 2020-03-24

**Authors:** Marie-Laëtitia Thézénas, Bianca De Leo, Alexis Laux-Biehlmann, Cemsel Bafligil, Bernd Elger, Thomas Tapmeier, Karl Morten, Nilufer Rahmioglu, Stephanie G. Dakin, Philip Charles, Fernando Estrada Martinez, Graham Steers, Oliver M. Fischer, Joerg Mueller, Holger Hess-Stumpp, Andreas Steinmeyer, Sanjiv Manek, Krina T. Zondervan, Stephen Kennedy, Christian M. Becker, Catherine Shang, Thomas M. Zollner, Benedikt M. Kessler, Udo Oppermann

**Affiliations:** 1 Target Discovery Institute, Nuffield Department of Medicine, University of Oxford, Oxford, OX3 7FZ UK; 2 Bayer AG, R&D, Gynaecological Therapies, 13342 Berlin, Germany; 3 Botnar Research Centre, NIHR Biomedical Research Unit Oxford, Nuffield Department of Musculoskeletal Sciences, University of Oxford, Oxford, OX3 7LD UK; 4 Endometriosis CaRe Centre, Nuffield Department of Obstetrics & Gynaecology, Oxford, UK; 5 Wellcome Trust Centre for Human Genetics, University of Oxford, Oxford, OX3 7BN UK; 6 Freiburg Institute for Advanced Studies (FRIAS), University of Freiburg, 79104 Freiburg, Germany

Correction to: *Scientific Reports* 10.1038/s41598-020-58362-3, published online 30 January 2020

In Figure 5A, the chemical structure is incorrect. As a result, the Figure legend,

“AOC3 inhibitor PXS-4681A shows analgesic effects in the endometriosis inoculation mouse model.

(**A**) Structure of AOC3 inhibitor PXS-4681A, orally administered BID at 2 mg/kg. (**B**) Unbound plasma levels of PXS-4681A (at 1-2-4 mg/kg). (**C**) Target engagement results (2 mg/kg). (**D**) Changes in H_2_O_2_ in plasma. (**E**) Plasma exposure of PXS-4681A at day 2. (**F**) Front/rear paw ratio measure using the dynamic weight bearing system indicating reduction of pain behaviour under treatment.”

should read:

“AOC3 inhibitor PXS-4681A shows analgesic effects in the endometriosis inoculation mouse model.

(**A**) Structure of AOC3 inhibitor PXS-4681A, orally administered BID at 2 mg/kg. The compound was synthesized according to literature procedures^37^ (**B**) Unbound plasma levels of PXS-4681A (at 1-2-4 mg/kg). (**C**) Target engagement results (2 mg/kg). (**D**) Changes in H_2_O_2_ in plasma. (**E**) Plasma exposure of PXS-4681A at day 2. (**F**) Front/rear paw ratio measure using the dynamic weight bearing system indicating reduction of pain behaviour under treatment.”

The correct Figure 5 and its accompanying legend appear below as Figure 1.

**Figure 1:**
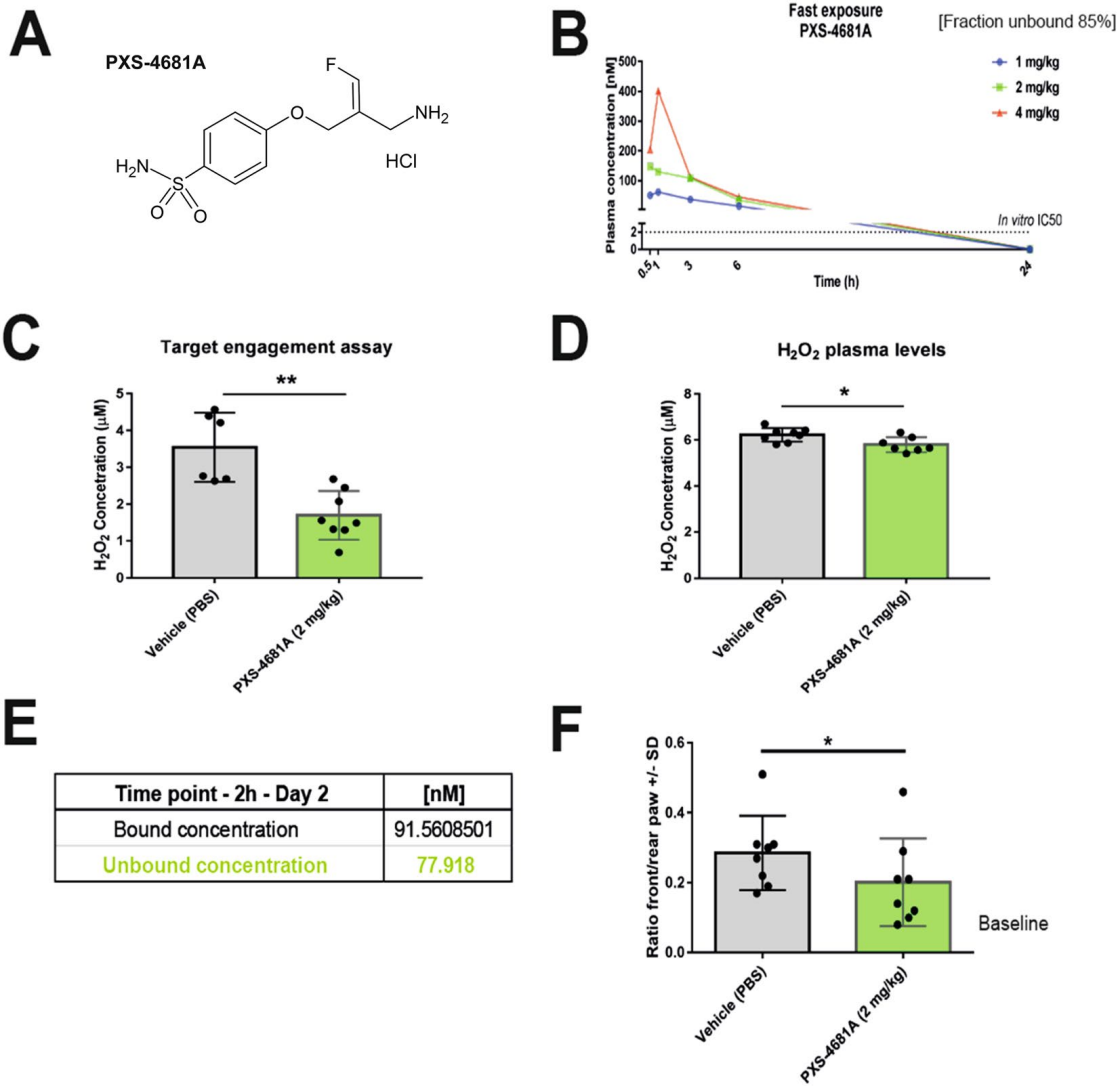
AOC3 inhibitor PXS-4681A shows analgesic effects in the endometriosis inoculation mouse model. (**A**) Structure of AOC3 inhibitor PXS-4681A, orally administered BID at 2 mg/kg. The compound was synthesized according to literature procedures^37^ (**B**) Unbound plasma levels of PXS-4681A (at 1-2-4 mg/kg). (**C**) Target engagement results (2 mg/kg). (**D**) Changes in H2O2 in plasma. (**E**) Plasma exposure of PXS-4681A at day 2. (**F**) Front/rear paw ratio measure using the dynamic weight bearing system indicating reduction of pain behaviour under treatment.

