# Supplementary material for: Amine oxidase 3 is a novel pro-inflammatory marker of oxidative stress in peritoneal endometriosis lesions
Source: Sci Rep. 2020 Jan 30;10:1495. doi: 10.1038/s41598-020-58362-3 (PMC6992811; doi:10.1038/s41598-020-58362-3)
Supplement: Supplementary file 1 — SI Appendix. [file 41598_2020_58362_MOESM1_ESM.docx]

**TITLE: Amine oxidase 3 is a novel pro-inflammatory marker of oxidative stress in peritoneal endometriosis lesions**

**AUTHORS:** Marie-Laëtitia Thézénas, Bianca De Leo, Alexis Laux-Biehlmann, Cemsel Bafligil, Bernd Elger, Thomas Tapmeier, Karl Morten, Nilufer Rahmioglu, Stephanie G Dakin, Philip Charles, Fernando Estrada Martinez, Graham Steers, Oliver M Fischer, Joerg Mueller, Holger Hess-Stumpp, Andreas Steinmeyer, Sanjeev Manek, Krina T Zondervan, Stephen Kennedy, Christian M Becker, Catherine Shang, Thomas M Zollner, Benedikt M Kessler, Udo Oppermann

**MATERIALS and METHODS**

***Patient samples*** – For the proteomics study matched eutopic endometrium and ectopic lesion tissue samples (n=6; stages 1-3) were collected on days 2-9 of the menstrual cycle and control eutopic tissues (n=5) were collected on days 4-11 of the menstrual cycle. All samples were collected from patients undergoing laparoscopy at the John Radcliffe Hospital, Oxford. In all cases diagnosis was confirmed surgically and by histology. Tissue from cases (n=6) and controls (n=5) from Oxford originated from the ENDOX study, which was approved by the local Research Ethics Committee (National Health Services (NHS) Research (NRES) Committee South Central-Oxford) (09/H0604/58). For the transcriptomics study, matched eutopic endometrium and ectopic lesion tissue samples (n=18; 11 stageI/II and 7 stage III/IV) were collected within the ENDOX study and were different to the proteomics cohort. Patients were not on hormone therapy for the past three months and had regular menstrual cycles. Of the 18 patients (transcriptomics), 13 were in secretory phase, 3 were in proliferative phase and 2 were in menstrual phase when samples were collected.

***Protein extraction and sample preparation*** *-* Tissue samples (~1mm^3^) were homogenised in RIPA buffer and protease inhibitor (Roche). 20 µg of each lysate was used for processing. Proteins were reduced with dithiothreitol followed by alkylation with iodoacetamide. Chloroform and methanol precipitation was used for buffer exchange and each pellet was resuspended solubilised. Trypsin (Promega) was added to each sample for overnight digestion at 37°C, 300 rpm. The samples were desalted using Sola cartridges (Waters) per the manufacturer’s instructions. In brief, the cartridges were conditioned with buffer A (1% CH_3_CN, 0.1% TFA in water) prior equilibration with buffer B (65% acetonitrile, 0.1% TFA in water). The acidified peptides were loaded onto the column, washed with buffer A and eluted with buffer B. The samples were dried by centrifugation in vacuo, resuspended in 1% acetonitrile, 0.1% TFA in water and stored at -20^o^C until analysis.

***Analysis by mass spectrometry*** *-* Peptides were analysed by liquid chromatography tandem mass spectrometry (LC-MS/MS) using a Q Exactive-High Field (HF) mass spectrometer (Thermo Scientiﬁc, Bremen, Germany) coupled to an ultra-high performance liquid chromatography system (Dionex Ultimate 3000 UHPLC, Thermo). The samples were loaded onto a PepMap RSLC column (C18, 2μm, 100A, 75μm x 50 cm) and analysed using a 60-minute linear gradient at a 250 nl/minute flow rate. The gradient used to elute the peptides started at 3 minutes with 2 % buffer B (0.1% TFA and 5% DMSO in CH_3_CN) increasing to 5% by 6 minutes, followed by an increase up to 35% by 63 minutes. The total length of the analysis was 100 minutes to allow column re-equilibration. The data were acquired with a resolution of 70,000 full-width at half maximum ion intensity with a mass/charge ratio of 400 and a lock mass enabled at 445.120025 m/z. Top 15 precursor ion selection, dynamic exclusion of 27 seconds and fragmentation were performed in higher-energy C-trap dissociation (HCD) mode with a normalised collision energy of 28. Raw MS data was analysed using MaxQuant (v1.3.1). Each sample was set as one experiment. The search settings were as follows: trypsin with two miss cleavages allowed, oxidation (M) and Deamidation (N, Q) were set as variable modifications and carbamidomethylation (C) as fixed modification. The data was searched against human protein sequences using the UPR_homoSapiens_20141015 fasta file along with the corresponding decoy revert database. Unique and razor peptides only were used for quantitation. The proteins identified in the search were processed using Perseus software (v15.6.0), which filtered contaminant and false positive identifications (decoys). After filtering, the remaining proteins were further investigated. The data were log2 transformed, and replicates were grouped into endometriosis ectopic, eutopic as well as eutopic control categories (SI Data 1). The missing values were replaced using a strategy that considers normal distribution of imputed values. Heat maps were created in Perseus using the Pearson Correlation algorithm applied after converting values to Z scores. Principal component analysis (PCA) was performed using Perseus software on all the proteins identified by LC-MS/MS. The PCA shows the replicates grouping based on the best combination of proteins enable to explain most of the variability of the data. For the proteomics data analysis (**SI Data I**) and the generation of the volcano plot shown in **Figure 3**, we used Perseus software by applying a parametric Student t-test (assuming a normal distribution of the quantitative mass spectrometry data) of a comparison between eutopic and ectopic samples (log2 of the difference in total intensities per protein) and using a Permutation FDR based correction for multiple testing ^1^. Pathway enrichment analysis of differentially expressed proteins or transcripts was performed using the Reactome application at the European Bioinformatics Institute. A binomial test was used to calculate the probability of over-representation of pathways, and p-values were corrected for multiple testing (Benjamini-Hochberg procedure).

**Transcriptome analysis of eutopic and ectopic lesions** - **Transcriptome analysis –** 36 samples (matched 18 eutopic endometrium and 18 endometrial lesions) were profiled using whole genome RNA sequencing. Sequencing depth was 36.5M per sample and ribo-deletion was used for extraction of mRNA. Reads were mapped (>90%) to transcriptome using HISAT2 ^2^. Gene count data was generated using the featureCount package in R, and processed using standard methods. Briefly, duplicate and multiply-mapped reads were excluded before read quality control (QC): filtering by number of reads mapping to all ensembl genes, transcripts per million (TPM), and % reads mapped to top 100 genes (complexity measure); visual inspection of diagnostic plots (# genes with >=TPM vs. Complexity, principal component analysis (PCA) and hierarchical cluster plots. Genes with count-per-million (CPM) >1 in 80% of the samples were included in the analysis (n=17,673). Data were variance stabilised using DESeq library in R ^3^. TMM normalisation was applied on the gene counts. To test expression differences between endometrium vs. endometrial lesions, generalised linear models (GLM) were fitted for each gene using edgeR in R ^4^. A false-discovery-rate (FDR) correction was applied based on the number of tests (N=17,673). A gene set connected to oxidative stress and lipid peroxidation (SI Data 2) was extracted using the GLAD4U search engine ^5^ and applied to the RNAseq dataset (SI Data 3). Differentially expressed genes were then analysed for pathway assignment using the Reactome application at EBI^6^.

***Processing of human endometrial tissues for immunohistochemical staining -*** Samples of human endometrial tissues were immersed in 10% buffered formalin for 24 hours at room temperature. After fixation, tissues were processed using a Leica ASP300S tissue processor and embedded in paraffin wax. Tissues were sectioned to 4μm using a rotary RM2135 microtome (Leica Microsystems Ltd) onto adhesive glass slides.

***Immunohistochemistry*** ***-*** For antigen retrieval, slides were baked at 60°C for 60 minutes and tissue sections were taken through deparaffinisation and target retrieval steps (high pH heat mediated antigen retrieval or at pH 6 using a citrate buffer) using an automated PT Link (Dako). Antibody staining was performed using the EnVision FLEX visualization system with an Autostainer Link 48 (Dako) using primary antibodies as detailed (SI Table 1). Antibody binding was visualized using FLEX 3,3’-diaminobenzidine (DAB) or in the case of CD68 AP (GBI Permanent Red Kit) substrate working solution and haematoxylin counterstain (Dako) as per protocols provided by the manufacturer. For negative controls the primary antibody was substituted for universal isotype control antibodies: cocktail of mouse IgG_1_, IgG_2a_, IgG_2_b, IgG_3_ and IgM (Dako) and rabbit immunoglobulin fraction of serum from non-immunised rabbits, solid phase absorbed (Dako). After staining, slides were taken through graded industrial methylated spirit and xylene and mounted in Pertex mounting medium (Histolab).

***Immunofluorescence for co-localization of PTGR and MSRB3 with macrophage markers -*** The protocol is adapted from ^7^. After antigen retrieval steps, tissues were blocked in 5% goat serum (Sigma) in PBS for 45 minutes in a humid chamber at RT. Sections were incubated with the primary antibody cocktail diluted in 5% normal goat serum in PBS for 2 hours at room temperature. Primary antibodies included CD68, CD163 PTGR1 and MSRB3 each diluted 1:200. Sections were washed 3 times with PBST and incubated in the secondary antibody cocktail (goat anti-mouse IgG1FITC Southern Biotech, goat anti-mouse IgG2a Alexa Fluor 568 and goat anti-rabbit IgG Alexa Fluor 633, Life Technologies) each diluted 1:200 in 5% normal equine serum (Sigma) in PBS for 2 hours. After washing, sections were incubated in 2μM POPO-1 nuclear counterstain (Life Technologies) diluted in PBS containing 0.05% Saponin (Sigma) for 20 minutes. Tissue auto fluorescence was quenched with a solution of 0.1% Sudan Black B (Applichem) for 5 minutes. Slides were mounted using fluorescent mounting medium (VectaShield), sealed and stored at 4°C until image acquisition. Immunofluorescence images were acquired on a Zeiss LSM 710 confocal microscope using a ×40 oil immersion objective (NA=1.3). The fluorophores POPO-1, Alexa Fluor 488, Alexa Fluor 568, and Alexa Fluor 633 were excited using the 405nm, 488nm, 561nm, and 633nm laser lines respectively. To minimize bleed-through, all channels were acquired sequentially. Averaging was set to 2 and the pinhole was set to approximately 1 Airy unit. Two-dimensional image reconstructions were created using ZEN 2009 (Zeiss).

**Cell culture** - Human peripheral blood mononuclear cells (PBMCs) were freshly isolated. Pro Leucosep-tubes were filled with 15 ml Ficoll-Paque and pushed down through the inserted frit by short centrifugation (30 sec 250 xg). The tubes were filled with 20 ml human blood and centrifuged 15 min at 800 xg. The plasma supernatant was discarded. Mononuclear cells from five Leucosep-tubes were transferred to 50 ml tubes that were centrifuged 10 min, 250 xg. Following supernatant withdrawal, the resulting pellet was resuspended and washed in PBS. The final pellet was resuspended in 6 ml cell culture medium (20% FCS). Cell viability was assessed using ApoLiveGlo.

**Effect of 4-HNE treatment on monocytic cells -** The human monocyte cell line THP-1 (ATCC) was grown in 1640 RPMI (Gibco, further adjusted, glucose 4.5g/L, HEPES 10mM, sodium pyruvate 1mM, foetal bovine serum (FBS) to 10% (v/v)) in the absence of antibiotics. Cells were incubated for 2hrs in media containing different concentrations of 4-HNE (Calbiochem) with and without FBS for 2 hrs, collected by centrifugation and washed with cold PBS. Cells were lysed in NP-40 extraction buffer (150mM NaCl, Triton X-100 (1%), Tris pH 8.0, complete mini protease inhibitors (Boehringer?)). Protein concentrations were determined using the BCA assay (Pierce) and 10µg of denatured protein were loaded per well on 4-20 %Tris/Glycine SDS/PAGE gels (Novex). Proteins were transferred by blotting onto PVDF membranes (Immobilon-FL, Millipore) and stained with Ponceau S. Resulting images were quantified using a G-box system (Syngene); membranes were blocked with 5% Blotto (Alpha Diagnostics) in TBS-T for 2 hrs at room temperature. Membranes were probed with an antibody to 4-HNE (Abcam ab46545 1/1000) to identify 4-HNE modified proteins. An anti-rabbit HRP secondary antibody (DAKO) diluted at 1/2000 in combination with chemilluminescence (Promega ECL) was used for detection and quantification using a G-box and gene tools software (Syngene). The 4-HNE signal was standardised relative to Ponceau S stained loading images. The effect of 4-HNE on THP-1 cell death was determined in 96 well plates after incubation with different concentrations of 4-HNE with and without serum for 24hrs. Following incubation a cell death assay using propidium iodide was used to quantify cell death relative to total cell number.

**Measurement of cytokine levels** - Cytokine levels in peritoneal fluid samples of endometriosis patients (n=69) and non-endometriotic controls (n=29) were measured using the V-PLEX Human Cytokine 36-Plex Kit (Meso Scale Discovery) according to the manufacturer’s instructions. Only samples that had not been diluted by washing during surgery were used. Cytokine data were analysed using SPSS (v22, IBM) and plotted in Prism (v6, Graph Pad, Inc.). Data from 3 technical replicates each were grouped by patient cycle phase and the amounts of cytokines between endometriosis patients and controls were compared using the non-parametric Mann-Whitney U-Test for independent samples. A p-value of <0.05 was considered significant.

***Inhibition of AOC3 in LPS-Induced Peritonitis model***

*Animals and treatment* **-** Animals were acclimatized for one week and maintained in a 12 h:12 h light:dark cycle with access to food and water *ad libitum*. All experiments were approved by Bayer AG and in accordance with policies and directives of LAGeSo (Landesamt für Gesundheit und Soziales Berlin; Germany). BALB/c female mice 8-10 weeks old and weighing approx. 20 g (Janvier Labs), were given i.p. injections of 5 mg/kg LPS (dissolved in PBS, serotype O111:B4; Sigma-Aldrich). 1h before LPS injection, animals were orally treated with 10 mg/kg and 30 mg/kg dissolved in PBS (16 animals/group). At 1h and 4h after LPS administration, peritoneal lavage, peritoneal cells and plasma were collected.

*Sample collection* **-** 3ml of ice-cold PBS (with 1% FCS) were injected into the peritoneal cavity of anaesthetised animals. After injection, mice were gently agitated to free cells and proteins into PBS solution, and approx. 2 mL of lavage were collected. The lavage was kept on ice until it was processed. The collected fluid was centrifuged at 900 g for 10 min at 4°C, the supernatant was stored at 80°C until measurement. Blood was drawn from the abdominal aorta of anesthetized animals, and plasma was collected and frozen until time of cytokine measurements. Mice were then culled by cervical dislocation. Protein concentrations in the peritoneal lavage were analysed using the Bio-Plex™ multiplex system (#M60-009RDPD, Bio-Plex Mouse Cytokine 23-plex Assay, Bio-Rad, Hercules, CA, USA) following the manufacturer`s instructions.

*Dynamic weight bearing evaluation* **-** The automated DWB device (Bioseb, Boulogne, France) comprises of a Plexiglas enclosure, a floor sensor detecting body pressure variation, and of a camera for animal position evaluation during the analysis ^8^. DWB recording took place 4h after i.p. LPS injection. Animals underwent to a habituation phase of 5 min followed by a recording phase of 5 min. The recordings were analysed with the DWB software (v1.4.1.28; Bioseb).

*Statistical Analysis* **-** Data were plotted as mean ± SD. When two groups were compared, the Mann–Whitney test was used to evaluate the significance. For analysis of more than two groups, the one-way ANOVA followed by Dunnett´s multiple comparisons test was performed. The Grubbs test was used to identify potential outliers. GraphPad Prism 7 (GraphPad Software Inc., La Jolla, CA, USA) was used for all analyses and statistical calculations.

**REFERENCES**

1 Tyanova, S. *et al.* The Perseus computational platform for comprehensive analysis of (prote)omics data. *Nat Methods* **13**, 731-740, doi:10.1038/nmeth.3901 (2016).

2 Pertea, M., Kim, D., Pertea, G. M., Leek, J. T. & Salzberg, S. L. Transcript-level expression analysis of RNA-seq experiments with HISAT, StringTie and Ballgown. *Nat Protoc* **11**, 1650-1667, doi:10.1038/nprot.2016.095 (2016).

3 Anders, S. & Huber, W. Differential expression analysis for sequence count data. *Genome Biol* **11**, R106, doi:10.1186/gb-2010-11-10-r106 (2010).

4 McCarthy, D. J., Chen, Y. & Smyth, G. K. Differential expression analysis of multifactor RNA-Seq experiments with respect to biological variation. *Nucleic Acids Res* **40**, 4288-4297, doi:10.1093/nar/gks042 (2012).

5 Jourquin, J., Duncan, D., Shi, Z. & Zhang, B. GLAD4U: deriving and prioritizing gene lists from PubMed literature. *BMC Genomics* **13 Suppl 8**, S20, doi:10.1186/1471-2164-13-S8-S20 (2012).

6 Fabregat, A. *et al.* The Reactome pathway Knowledgebase. *Nucleic Acids Res* **44**, D481-487, doi:10.1093/nar/gkv1351 (2016).

7 Dakin, S. G. *et al.* Inflammation activation and resolution in human tendon disease. *Sci Transl Med* **7**, 311ra173, doi:10.1126/scitranslmed.aac4269 (2015).

8 Robinson, I., Sargent, B. & Hatcher, J. P. Use of dynamic weight bearing as a novel end-point for the assessment of Freund's Complete Adjuvant induced hypersensitivity in mice. *Neurosci Lett* **524**, 107-110, doi:10.1016/j.neulet.2012.07.017 (2012).
